# Late gadolinium enhancement cardiovascular magnetic resonance with generative artificial intelligence

**DOI:** 10.1016/j.jocmr.2024.101127

**Published:** 2024-11-28

**Authors:** Omer Burak Demirel, Fahime Ghanbari, Christopher W. Hoeger, Connie W. Tsao, Adele Carty, Long H. Ngo, Patrick Pierce, Scott Johnson, Kathryn Arcand, Jordan Street, Jennifer Rodriguez, Tess E. Wallace, Kelvin Chow, Warren J. Manning, Reza Nezafat

**Affiliations:** a Department of Medicine (Cardiovascular Division) and Harvard Medical School, Boston, Massachusetts, USA; b Department of Radiology, Beth Israel Deaconess Medical Center and Harvard Medical School, Boston, Massachusetts, USA; c Siemens Medical Solutions USA, Inc., Boston, Massachusetts, USA; d Cardiovascular MR R&D, Siemens Healthcare Ltd., Calgary, Alberta, Canada

**Keywords:** Late gadolinium enhancement, Highly accelerated, Deep learning

## Abstract

**Background:**

Late gadolinium enhancement (LGE) cardiovascular magnetic resonance (CMR) imaging enables imaging of scar/fibrosis and is a cornerstone of most CMR imaging protocols. CMR imaging can benefit from image acceleration; however, image acceleration in LGE remains challenging due to its limited signal-to-noise ratio. In this study, we sought to evaluate a rapid two-dimensional (2D) LGE imaging protocol using a generative artificial intelligence (AI) algorithm with inline reconstruction.

**Methods:**

A generative AI-based image enhancement was used to improve the sharpness of 2D LGE images acquired with low spatial resolution in the phase-encode direction. The generative AI model is an image enhancement technique built on the enhanced super-resolution generative adversarial network. The model was trained using balanced steady-state free-precession cine images, readily used for LGE without additional training. The model was implemented inline, allowing the reconstruction of images on the scanner console. We prospectively enrolled 100 patients (55 ± 14 years, 72 males) referred for clinical CMR at 3T. We collected three sets of LGE images in each subject, with in-plane spatial resolutions of 1.5 × 1.5-3-6 mm^2^. The generative AI model enhanced in-plane resolution to 1.5 × 1.5 mm^2^ from the low-resolution counterparts. Images were compared using a blur metric, quantifying the perceived image sharpness (0 = sharpest, 1 = blurriest). LGE image sharpness (using a 5-point scale) was assessed by three independent readers.

**Results:**

The scan times for the three imaging sets were 15 ± 3, 9 ± 2, and 6 ± 1 s, with inline generative AI-based images reconstructed time of ∼37 ms. The generative AI-based model improved visual image sharpness, resulting in lower blur metric compared to low-resolution counterparts (AI-enhanced from 1.5 × 3 mm^2^ resolution: 0.3 ± 0.03 vs 0.35 ± 0.03, *P* < 0.01). Meanwhile, AI-enhanced images from 1.5 × 3 mm^2^ resolution and original LGE images showed similar blur metric (0.30 ± 0.03 vs 0.31 ± 0.03, *P* = 1.0) Additionally, there was an overall 18% improvement in image sharpness between AI-enhanced images from 1.5 × 3 mm^2^ resolution and original LGE images in the subjective blurriness score (*P* < 0.01).

**Conclusion:**

The generative AI-based model enhances the image quality of 2D LGE images while reducing the scan time and preserving imaging sharpness. Further evaluation in a large cohort is needed to assess the clinical utility of AI-enhanced LGE images for scar evaluation, as this proof-of-concept study does not provide evidence of an impact on diagnosis.

## Graphical abstract

**Figure:**
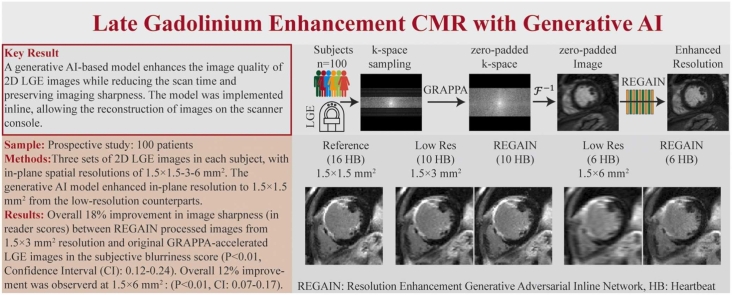

## 1 Background

Evaluation of myocardial scar remains a major indication for patients referred for cardiovascular magnetic resonance (CMR) [1]. Late gadolinium enhancement (LGE) is the gold-standard imaging technique to evaluate scar presence and burden using an inversion recovery-based sequence [2]. Over the past two decades, there have been steady technical improvements in LGE imaging, including phase-sensitive inversion recovery LGE [3], dark/gray-blood LGE [4], [5], [6], [7], [8], [9], single-shot LGE [10], [11], and three-dimensional (3D) LGE [12], [13], [14]. These advances have improved the diagnostic confidence of LGE imaging, sensitivity/specificity in detecting scars, spatial resolution, and image quality in patients with poor electrocardiography (ECG) gating or breath-holding [15]. However, despite progress in image acceleration to reduce scan time, image acceleration in LGE remains challenging due to the intrinsic low signal-to-noise ratio (SNR).

Image acceleration using parallel imaging is commonly used in CMR imaging to reduce the scan time [16], [17]. However, the considerable SNR penalty of parallel imaging with high acceleration rates limits its use in two-dimensional (2D) LGE imaging [18]. Meanwhile, compressed sensing (CS) enables a higher image acceleration factor than parallel imaging and has better artifact suppression [19]. However, it performs well in sparse multi-dimensional data (e.g., 3D LGE [13], [14], [20], [21], [22]) and has not been successful in 2D LGE. Therefore, despite the need to reduce the 2D LGE scan time, there has been very limited progress.

Artificial intelligence (AI)-based image acceleration techniques have recently been introduced in CMR with promising initial results [23], [24], [25], [26], [27], [28]. These techniques can be broadly categorized into image denoising, image reconstruction, and image enhancement. Image denoising is utilized for SNR, compensating for lost quality due to acceleration factors or inherently low SNR in acquisitions [29], [30]. Image reconstruction is performed in either k-space to k-space/image models [31], [32], [33] or image-to-image models [34], [35], [36], [37], [38], [39], [40], [41]. For the former convolutional neural networks (CNNs) are trained to interpolate missing k-space samples from raw multi-coil data. In the latter, CNNs are trained for de-aliasing corrupted images resulting from undersampling, using fully sampled images as reference pairs. Alternatively, image reconstruction is iteratively performed to reconstruct raw multi-channel data by alternating between data consistency and regularization steps [42], [43], [44], [45], [46], [47], [48]. Conversely, image enhancement, also known as super-resolution, aims to enhance or restore the spatial resolution of low-resolution images [28], [49], [50], [51], [52], [53], [54], [55].

Unlike parallel imaging, CS or AI-based image reconstruction techniques, which achieve acceleration through equispaced or random undersampling, image enhancement acquires truncated k-space data. This requires fewer k-space lines, resulting in an anisotropic low-resolution image. It also eliminates the need for sequence modifications or advanced sampling strategies required by CS-based methods and avoids aliasing artifacts. Additionally, image enhancement via generative adversarial networks extends beyond mere training to minimize differences between low-resolution and reference high-resolution images. Rather, its objective is to generate images that closely resemble those from the target dataset, exhibiting sharper details and edges, thus approaching the quality of true high-resolution images [56].

In this study, we sought to develop and evaluate a 2D LGE imaging protocol using a generative AI-based image enhancement model. The accelerated LGE imaging via truncated k-space in phase-encode (PE) direction was performed using a commercially available sequence without modification. The open-source trained model was integrated into a clinical CMR scanner for rapid inline processing of accelerated LGE. However, as this is a proof-of-concept study, no current evidence indicates an impact on diagnosis.

## 2 Methods

Imaging was performed on a 3T magnetic resonance imaging (MRI) system (MAGNETOM Vida, Siemens Healthineers, Forchheim, Germany) using a commercial 18-channel phase body array coil and posterior spine array coils. We prospectively recruited patients referred for clinical CMR exam for myocardial scar evaluation. Patients were approached before the CMR exam for willingness to allow additional research scanning at the end of their clinical exam, which added <5 min to their clinical exam. This is a Health Insurance Portability and Accountability Act (HIPAA)-compliant study approved by our local institutional review board. Written informed consent was collected from each subject before the investigation.

### 2.1 Resolution enhancement generative adversarial inline neural network

We implemented a research inline algorithm for LGE reconstruction using our previously reported resolution enhancement generative adversarial inline neural network (REGAIN) image enhancement technique for cardiac cine [50]. REGAIN [50] is a deep learning (DL) image enhancement technique built on the enhanced super-resolution generative adversarial network (ESRGAN) [56]. Like in ESRGAN, the discriminator of REGAIN learns to differentiate the degree of realism between two images rather than making a binary determination of real or fake for individual images. In the meantime, densely connected residual-in-residual block convolutions were employed in its generator. Expanding upon the ESRGAN framework, REGAIN incorporates $l_{1}$ fast-Fourier transform as an additional constraint in its loss function.

**Fig. 1:**
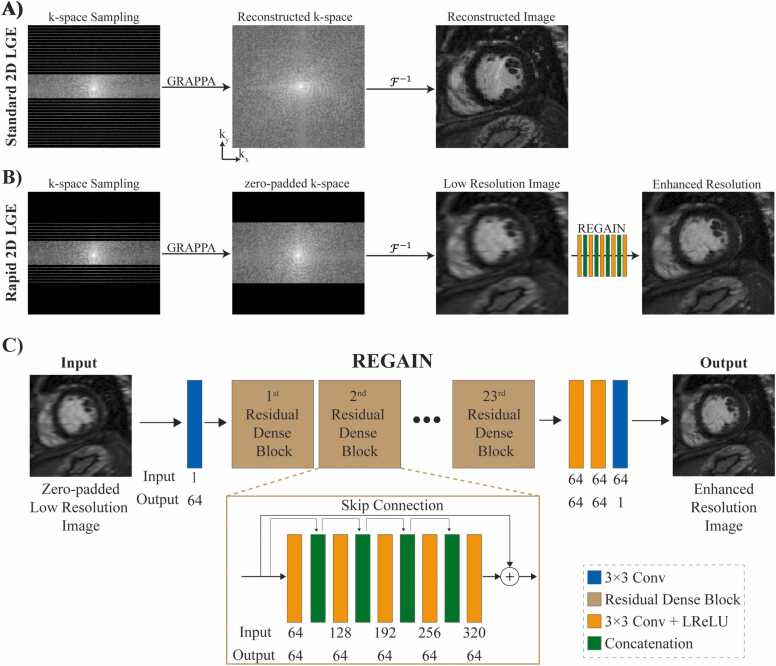
Schematic of k-space sampling and reconstruction of accelerated LGE with (A) GRAPPA and (B) GRAPPA followed by REGAIN image enhancement. In conventional accelerated 2D LGE imaging (A), k-space is sampled using a uniform undersampling with a fully sampled center line (i.e., ACS lines). Images are then reconstructed using standard GRAPPA reconstruction. In the REGAIN approach, images with lower spatial resolution along the phase-encoding direction are prescribed, reducing scan time. This results in reducing k_y_^max^ values. Standard GRAPPA reconstruction is then used to reconstruct images with a lower spatial resolution. The low-resolution images are then fed into the REGAIN image enhancement model to enhance the image quality. (C) REGAIN generator network consists of 23 residual dense blocks, each having 3 × 3 convolutions, leaky rectified linear unit (LReLU), and concatenation of the features in channel dimension. *LGE* late gadolinium enhancement, *GRAPPA* generalized autocalibrating partially parallel acquisitions, *2D* two-dimensional, *REGAIN* resolution enhancement generative adversarial inline neural network, *ACS* auto-calibrated lines

The REGAIN generator network consists of 23 residual dense blocks, each incorporating convolution layers followed by a non-linear activation via leaky rectified linear unit (LReLU) and a feature concatenation in the channel dimension (Fig. 1). Concurrently, the discriminator (Additional File 1: Fig. S1) is trained using six discriminator blocks, each integrating convolution layers, batch normalization, and LReLU. REGAIN was trained on ECG-gated segmented cine images by discarding 25%–50% of outer PE lines and targeted to high-resolution images with pixel loss, relativistic average generative adversarial loss, visual geometry group loss, and $l_{1}$ fast-Fourier transform loss. REGAIN was trained on data from 1616 participants (age 56 ± 16 years, 920 male) who underwent clinical CMR at Beth Israel Deaconess Medical Center between October 2018 and August 2021. The training used short-axis ECG-gated cine acquisitions with the following parameters: repetition time (TR)/echo time (TE) = 2.9–3.3/1.3–1.5 ms, flip angle = 37–68°, field of view (FOV) = 184–546 × 184–546 mm^2^, acquisition matrix = 132–210 × 132–210, in-plane resolution = 1.4–2.6 × 1.4–2.6 mm^2^, and slice thickness = 8–8.5 mm. To simulate low-spatial-resolution images during training, low-resolution images were synthesized by discarding 25%–50% of the outer phase-encoding lines while maintaining the data in the readout direction. REGAIN was integrated inline on the scanner using the Siemens framework for image reconstruction (FIRE) prototype [57] and was rigorously evaluated for cardiac cine imaging [50]. No additional training was performed to adapt the model for LGE image enhancement. Instead, the pre-trained model from the original REGAIN implementation, trained using low-resolution cine images, was used.

The reconstruction pipeline involves initially acquiring an anisotropic low-resolution LGE with a k-space undersampling pattern designed for generalized autocalibrating partially parallel acquisitions (GRAPPA) reconstruction. This center portion of the k-space, corresponding to a low-resolution LGE image, is then reconstructed, followed by zero-padded to match the matrix size of a high-resolution image followed by REGAIN processing (Fig. 1B). Unlike conventional methods that accelerate the full extent of the PE direction using GRAPPA (Fig. 1A), REGAIN utilizes GRAPPA acceleration up to a predetermined low-resolution level, which the operator sets via the phase resolution. After the acquisition, the low-resolution images were initially reconstructed by the vendor-provided image calculation environment (ICE) and then transmitted to an external server via FIRE emitter [57]. Subsequently, REGAIN implementation on the server improves the image sharpness and sends back the enhanced images through the FIRE injector to be displayed on the scanner console. The details of the REGAIN architecture, training, and inline integration are given in Additional File 1.

### 2.2 Study protocol

Three sets of 2D LGE images with different spatial resolutions were collected in each patient: (a) 1.8-fold accelerated LGE with spatial resolution = 1.5 × 1.5 mm^2^ reconstructed using inline GRAPPA reconstruction, (b) LGE with 1.5 × 3 mm^2^ spatial resolution (i.e., 3.3-fold acceleration) reconstructed into 1.5 × 1.5 mm^2^ using REGAIN, and (c) LGE with 1.5 × 6 mm^2^ spatial resolution (i.e., 5.7-fold acceleration) reconstructed into 1.5 × 1.5 mm^2^ using REGAIN. The reported acceleration takes into account the number of auto-calibration signal(ACS) lines (e.g., 1.8-fold acceleration is prescribed as GRAPPA rate 2 with an additional 24 ACS lines). 2D LGE images were collected using a GRAPPA acceleration factor of 2 with 24 ACS lines and an 8 mm slice thickness. Data collection was performed every other heartbeat (2RR), allowing signal recovery. The imaging parameters (Table 1) were fixed across all three scans, except for the number of PE lines 240, 120, and 60, resulting in 16, 10, and 6 heartbeats, respectively. Imaging was performed on a single mid-left ventricular short-axis slice to reduce the additional research scan time and not modify scanning time slots. Baseline clinical characteristics and CMR data of the 100 subjects are given in Table 2. LGE images were acquired 16 ± 5 min after a bolus infusion of 0.01 mmol/kg body weight Gadobutrol [Gadavist] (Bayer Healthcare Pharmaceuticals, Berlin, Germany). The order of acquisitions was not randomized, with the 5.7-fold acquisition performed first, followed by the 3.3-fold, and the 1.8-fold acquisition last.

**Table 1:** Imaging parameters.

	First acquisition 1.8-fold acceleration	Second acquisition 3.3-fold acceleration	Third acquisition 5.7-fold acceleration
Number of heartbeats	16	10	6
Number of triggers	8	5	3
Resolution (mm^2^)	1.5 × 1.5	1.5 × 3	1.5 × 6
Phase encode (PE) lines	240	120	60
Phase resolution (%)	100	50	25
Field of view (FOV) (mm^2^)	360 × 360	360 × 360	360 × 360
Phase FOV (mm^2^)	100	100	100
Echo time (ms)	2.5	2.5	2.5
Repetition time (ms)	5.6	5.6	5.6
Flip angle (°)	20	20	20
Slice thickness (mm)	8	8	8
Auto calibration signal lines	24	24	24
In-plane acceleration	2	2	2
Number of acquired PE lines	108	72	42
Overall acceleration	1.8	3.3	5.7

Continuous data are presented as mean.

**Table 2:** Clinical characteristics and baseline CMR data.

	All patients (n = 100)
Baselines clinical	
Age (years)	55 ± 14
Male n (%)	72 (72)
Height (cm)	173 ± 10
Weight (kg)	83 ± 18
BMI (kg/m^2^)	28 ± 5
BSA (m^2^)	1.98 ± 0.3
HR (bpm)	72 ± 12
Systolic BP (mmHg)	127 ± 19
Diastolic BP (mmHg)	73 ± 13
Baselines CMR	
LV	
EDV (mL)	183 ± 53
ESV (mL)	83 ± 42
EF (%)	56 ± 11
LV Mass (g)	117 ± 42
RV	
EDV (mL)	166 ± 45
ESV (mL)	74 ± 27
EF (%)	56 ± 8

Continuous data are shown as mean ± standard deviation, categorical data as number (%)

*BMI* body mass index*, BSA* body surface area, *HR* heart rate*, BP* blood pressure*, CMR* cardiovascular magnetic resonance*, LV* left ventricle*, EDV* end-diastolic volume*, ESV* end-systolic volume*, EF* ejection fraction*, RV* right ventricle

Values represent mean +- standard deviation.

### 2.3 Image analysis

All images were reconstructed inline and readily available as a new digital imaging and communications in medicine (DICOM) series immediately after data acquisition. Quantitative analysis of image sharpness was evaluated using a blur metric [58]. This method considers how our eyes perceive blur and operates on the principle that when a sharp image is blurred with a low-pass filter, the surrounding pixels undergo significant alterations. Conversely, when a blurred image is further blurred, the changes in neighboring pixels are relatively minor. Blur metric first low-pass filters the original image along vertical and horizontal axes and analyzes the resultant variations in neighboring pixel values. Then, it calculates the difference in variations as absolute differences between the blurred images and the input image. This is followed by the sum of coefficients, which compares the variations of these differences. These are then aggregated and normalized to produce a blur metric ranging from 0 to 1, with 0 representing minimal perceived blur (sharper images) and 1 signifying substantial blur. This approach to image blur assessment is detailed in Additional File 1 and depicted by comparing a sharp and a blurry image in Additional File 1: Figs. S2A and S2B.

Image analysis was conducted using cvi42 (v5.11.1, Circle Cardiovascular Imaging, Calgary, Alberta, Canada). To assess potential artefactual enhancement in REGAIN-processed images, a clinical CMR reader (F.G.: 8 years of experience in clinical CMR) compared the REGAIN-processed images with low-resolution counterparts and reference images. The comparison was based on visual differences, specifically evaluating two scenarios: (i) no artefactual enhancement with REGAIN when no scar was present in the reference or low-resolution image, and (ii) no artefactual removal of the scar with REGAIN when a scar was present in the reference or low-resolution image. The data were presented side by side using cvi42 software, and a binary analysis (0: no artefactual enhancement, 1: artefactual enhancement) was performed to evaluate whether the REGAIN images showed noticeable differences from the reference or low-resolution images.

LGE images were independently reviewed for perceived blurriness and quality by three experienced readers (F.G: 8 years of experience in clinical CMR, C.W.H: 2 years, C.W.T: 15 years) using cvi42 software. All LGE images were anonymized, and readers were blinded to image acquisition/reconstruction techniques. Each reader independently evaluated images on a subjective 5-point Likert scale for blurriness (1 = no blurriness, 2 = minimal, 3 = moderate, 4 = severe, 5 = non-diagnostic) using cvi42 software. Diagnostic quality was evaluated on a subjective 3-point Likert scale (1 = diagnostic, 2 = non-confident, 3 = non-diagnostic). One of the co-authors (F.G: 8 years of experience in clinical CMR) additionally evaluated scar transmurality using cvi42 software with the following scale: 1 = <25%, 2 = 26%–50%, 3 = >50%.

### 2.4 Statistical analyses

A one-way (analysis of variance) ANOVA with subsequent paired Student *t*-test was used to evaluate the blur metric, which was used for quantitative analysis of image sharpness. Bonferroni correction was applied for pairwise tests with significance levels of 0.01. Intraclass correlation coefficients (ICCs) were used to assess consistency among observers with the following criteria: <0.2 = slight agreement, 0.21–0.40 = fair agreement, 0.41–0.60 = moderate agreement, 0.61–0.80 = good agreement, 0.81–1.00 = excellent agreement. All analyses were conducted using a two-tailed approach, and statistically significant differences were considered if α < 0.05. MATLAB (R2023b, MathWorks, Natick, Massachusetts) was used for ANOVA and ICC.

To assess whether the AI algorithm enhanced 2D LGE image sharpness and diagnostic quality, we calculated the difference in perceived blur and diagnostic quality scores between low-resolution images and their REGAIN-processed counterparts. Similarly, REGAIN-processed images were compared with 1.8-fold GRAPPA-accelerated LGE images. The differences were then classified into binary categories where ≥1 represents improvement in image sharpness and diagnostic quality, and <1 represents no improvement in image sharpness and diagnostic quality. A linear mixed model for repeated measures was fit to estimate the percent improvement between images, using a compound symmetry covariance structure and adjusted for reader to control for reader effect. Tests of significance were two-sided and assessed at α < 0.05. Statistical analyses were completed using SAS (9.4, SAS Institute, Inc., Cary, North Carolina).

## 3 Results

**Fig. 2:**
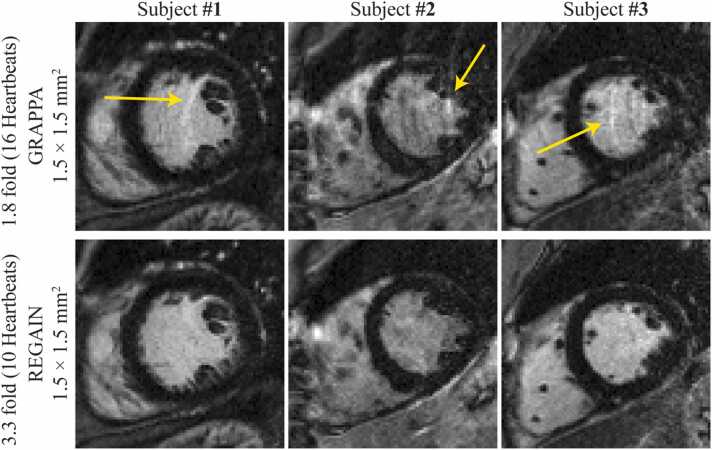
Representative LGE images from 3 participants with 16 heartbeat acquisition at 1.8-fold GRAPPA acceleration and 10 heartbeat acquisition using 3.3-fold REGAIN acceleration. In the standard acquisition requiring 16 heartbeats, respiratory motion resulted in ghosting artifacts (yellow arrows), which are not visible in shorter LGE scans. *LGE* late gadolinium enhancement, *GRAPPA* generalized autocalibrating partially parallel acquisitions, *REGAIN* resolution enhancement generative adversarial inline neural network

We successfully recruited 100 patients (55 ± 14 years, 72 males) referred for a clinical CMR exam for LGE imaging. The patient characteristics are summarized in Table 3 with 21 patients exhibiting LGE and 79 patients without LGE. Among the 21 patients with LGE, only 8 had subendocardial scars, allowing for a transmurality assessment. These scores are provided in Additional file 1: Table S1. All 100 participants, each undergoing three short-axis scans with different resolutions, were included in the study, and the low-latency reconstruction time was ∼37 ms per image [50]. All images were successfully reconstructed inline on the scanner console, and there was no failure. The measured scan time was 15 ±3 s, 9 ± 2 s, and 6 ± 1 s for 1.8-fold, 3.3-fold, and 5.7-fold accelerated acquisitions, respectively. The scan time varied between subjects because of heart rate. Although patients were instructed to hold their breath, we observed respiratory motion artifacts in images with longer scan times (Fig. 2).

**Table 3:** Patient characteristics.

Indication for CMR	n = 100
Cardiomyopathy	72
LV hypertrophy	8
Arrhythmia substrate	4
Pericarditis	2
Myocarditis	5
Coronary artery disease	3
Amyloid	1
Cardiac sarcoid	3
Valvulopathy	2
LGE conclusion	n = 21
Subendocardial LGE	8
Midwall LGE	7
Subepicardial LGE	3
Pericardial thickness	2
Diffuse LGE	1

*CMR* cardiovascular magnetic resonance*, LV* left ventricle*, LGE* late gadolinium enhancement

Values represent number of subject.For the rest of the tables, we cannot add any footnote since the data is not number of cases or mean or medians.

Image analysis did not find artefactual enhancement in any of the REGAIN-processed images. Additionally, scar transmurality did not show a difference between the 1.8-fold GRAPPA-accelerated, 3.3-fold low-resolution, 3.3-fold REGAIN-accelerated, 5.7-fold low-resolution, and 5.7-fold REGAIN-accelerated LGE images, with a mean value of 2.62 ± 0.48 for all methods.

**Fig. 3:**
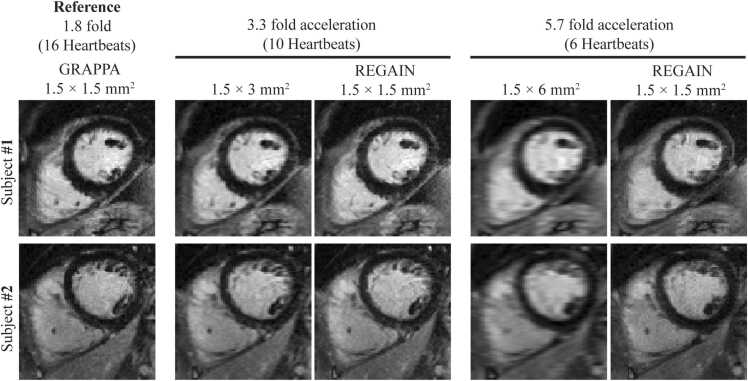
Representative LGE images from two subjects showing 1.8-fold accelerated GRAPPA with 1.5 × 1.5 mm^2^ resolution acquired in ∼15-second (16 heartbeats), 3.3-fold acceleration at ∼9-second (10 heartbeats) acquisition (1.5 × 3 mm^2^) with REGAIN enhancement, 5.7-fold acceleration at ∼6-second (6 heartbeats) acquisition (1.5 × 6 mm^2^) and with REGAIN enhancement. REGAIN enhances the image quality at both acceleration factors and preserves the structural details. *LGE* late gadolinium enhancement, *GRAPPA* generalized autocalibrating partially parallel acquisitions, *REGAIN* resolution enhancement generative adversarial inline neural network

**Fig. 4:**
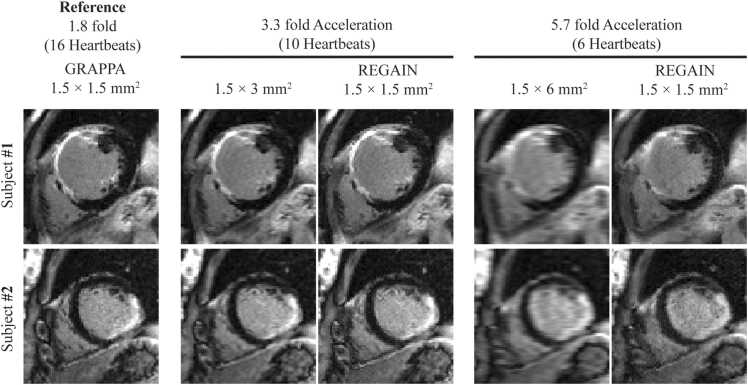
Representative LGE images from two participants with subendocardial scars on LGE. REGAIN improves the image quality of low-resolution images at both 3.3- and 5.7-fold accelerated images. There is an overall image quality loss at a higher acceleration rate when compared to the reference image; however, REGAIN enhancement improves image quality at both rates. *LGE* late gadolinium enhancement, *GRAPPA* generalized autocalibrating partially parallel acquisitions, *REGAIN* resolution enhancement generative adversarial inline neural network

**Fig. 5:**
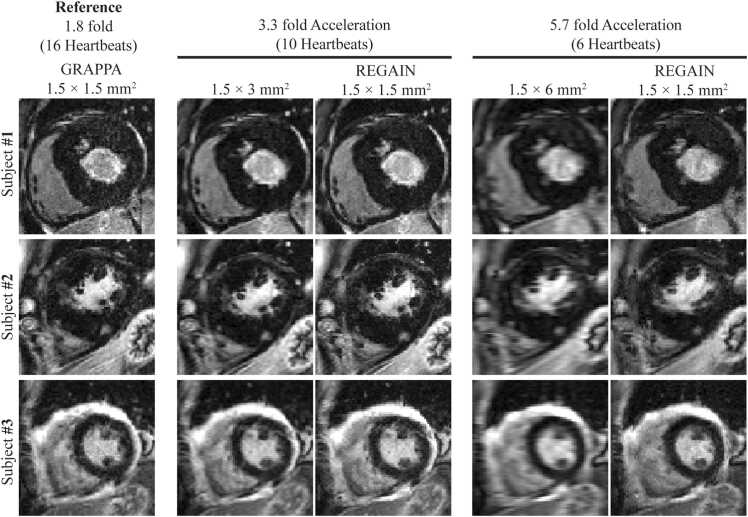
Representative LGE images from two patients with non-ischemic cardiomyopathies and one with pericarditis. REGAIN enhances myocardial scar and increases the visibility of the pericardial enhancement. *LGE* late gadolinium enhancement, *GRAPPA* generalized autocalibrating partially parallel acquisitions, *REGAIN* resolution enhancement generative adversarial inline neural network

Image quality was degraded with lower spatial resolution acquisition. LGE images with a prescribed in-plane resolution of 1.5 × 6 mm^2^ exhibited significant blurring (Fig. 3). REGAIN processing visually improved image sharpness, with more pronounced improvements at a higher acceleration rate (Fig. 3). There was no artifactual enhancement in any LGE images processed with REGAIN. Fig. 4 showed LGE images of two patients with subendocardial myocardial scars in the anterior septal/anterior wall and mid-inferior/inferolateral walls. REGAIN-enhanced scar details at 3.3-fold acceleration and preserved the extent of the scar compared with 1.8-fold acceleration. However, at a 5.7-fold acceleration, while REGAIN still improved image quality compared to the low-resolution counterpart, it did not fully preserve the scar architecture/extent, resulting in visual loss of details. LGE images in example patients with non-ischemic cardiomyopathies were shown in Fig. 5. In all cases, REGAIN improved visualization of scar or pericardial enhancement compared to low-resolution LGE images.

**Fig. 6:**
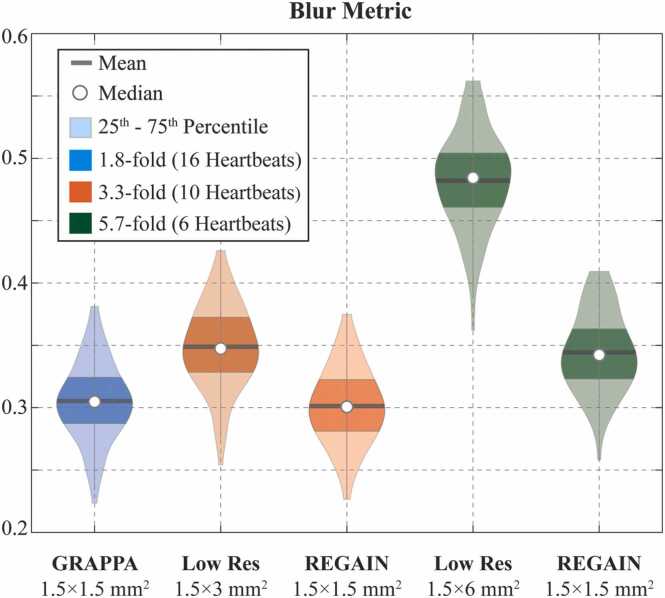
Blur metric results with violin plots for 1.8-fold GRAPPA-accelerated scan, 3.3- and 5.7-fold accelerations, and their REGAIN-enhanced images. Blur metric is defined as no-reference perceived blurriness that calculates the difference of the intensity variations between neighboring pixels of the image of interest and its low-pass filtered version. By looking at how much neighboring pixels change, the blur metric calculates a score ranging from 0 (no blur) to 1. REGAIN at both acceleration rates showed a smaller blur metric, indicating sharper image quality. REGAIN at 3.3-fold acceleration improved upon zero-padded LGE images (*P* < 0.01), meanwhile showing similar sharpness compared with 1.8-fold GRAPPA-accelerated LGE images (*P* = 1.0). *LGE* late gadolinium enhancement, *GRAPPA* generalized autocalibrating partially parallel acquisitions, *REGAIN* resolution enhancement generative adversarial inline neural network

Fig. 6 depicts violin plots for the blur metric. The 3.3-fold REGAIN-accelerated LGE images had higher sharpness compared to zero-padded counterparts (0.30 ± 0.03 vs 0.35 ± 0.03, respectively, *P* < 0.01) and had similar sharpness compared with 1.8-fold GRAPPA-accelerated LGE images (0.30 ± 0.03 vs 0.31 ± 0.03, respectively, *P* = 1.0). The 5.7-fold REGAIN-accelerated LGE images had higher sharpness compared with zero-padded counterparts (0.35 ± 0.03 vs 0.48 ± 0.04, respectively, *P* < 0.01) yet slightly lower sharpness compared with 1.8-fold GRAPPA-accelerated LGE images (0.35 ± 0.03 vs 0.31 ± 0.03, respectively, *P*<0.01). Furthermore, at 5.7-fold acceleration, zero-padded low-resolution images had the lowest sharpness compared with 1.8-fold GRAPPA-accelerated LGE images (0.48 ± 0.04 vs 0.31 ± 0.03, respectively, *P* < 0.01).

**Fig. 7:**
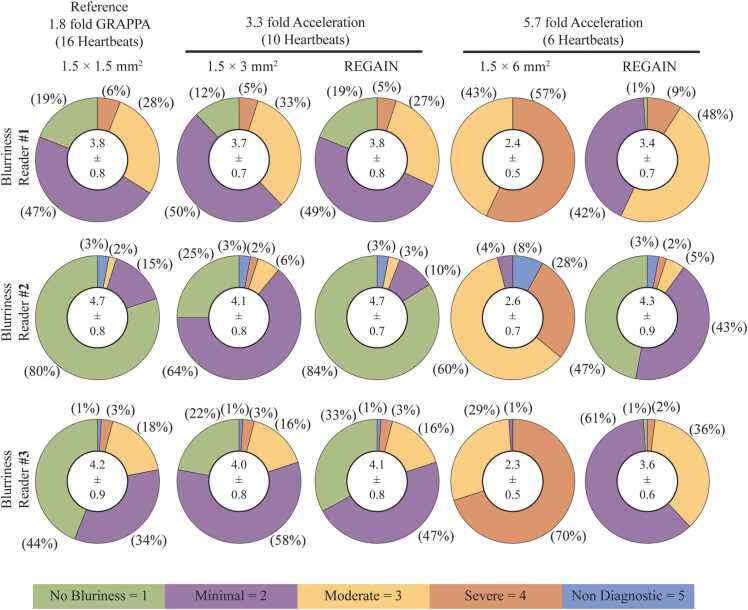
Subjective evaluation of perceived image blurriness (1 = no blurriness, 5 = non-diagnostic). Mean scores and standard deviations are depicted in the white circles, while percentages of no blur, minimal, moderate, severe, and non-diagnostic cases are illustrated in the pie charts. In 1.8-fold GRAPPA reconstructed images, percentages of scans with no blurriness were 19%, 80%, and 44% for the three readers. These percentages remained comparable in 3.3-fold REGAIN-processed images (19%, 84%, and 33%, respectively) but were lower in 3.3-fold accelerated images (12%, 25%, and 22%, respectively). There were no minimal blurring cases, according to the first reader, in 5.7-fold accelerated images, which was increased to 42% in 5.7-fold REGAIN-processed images. The second reader noted an increase from 4% to 43% in minimal blurring cases, while the third reader observed an increase from 1% to 61%. *GRAPPA* generalized autocalibrating partially parallel acquisitions, *REGAIN* resolution enhancement generative adversarial inline neural network

LGE images (a total of 300 imaging datasets) were subjectively evaluated by three independent readers for blurriness (Fig. 7). According to three readers, 19%, 80%, and 44% of scans were graded as having no blurriness (i.e., score = 1) in 1.8-fold GRAPPA reconstructed images. These percentages were comparable in 3.3-fold REGAIN-processed images, with 19%, 84%, and 33%, respectively, as assessed by the three readers, and lower in 3.3-fold accelerated images, with 12%, 25%, and 22%, respectively. Furthermore, there were no minimal blurring cases, according to the first reader, in 5.7-fold accelerated images, which increased to 42% in 5.7-fold REGAIN-processed images. Similarly, the number of minimal blurring cases increased from 4% to 43%, according to the second reader, and from 1% to 61%, according to the third reader.

**Fig. 8:**
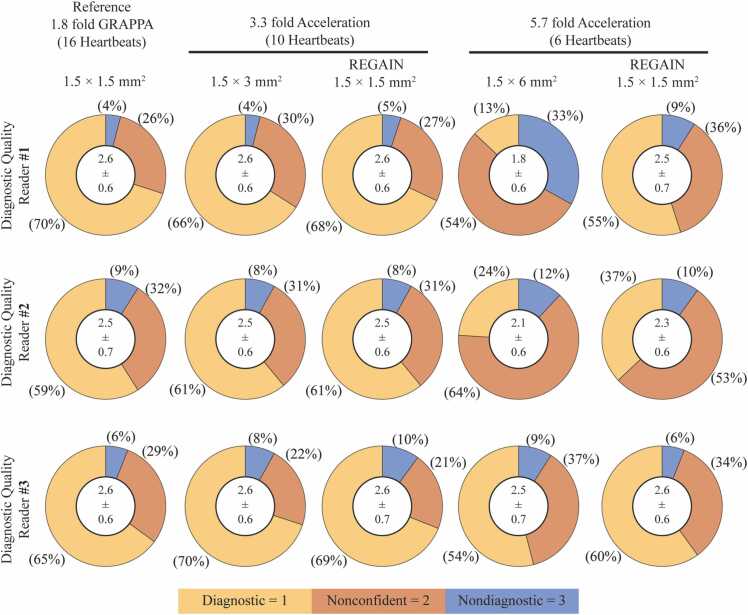
Subjective evaluation of diagnostic quality. Subjective evaluation of diagnostic quality is depicted through mean scores and standard deviations in the white circles, while percentages of diagnostic, non-confident, and non-diagnostic cases are illustrated in the pie charts. In 1.8-fold GRAPPA reconstructed images, the percentages of scans with diagnostic quality were 70%, 59%, and 65% for the three readers. These percentages remained comparable in 3.3-fold REGAIN-processed images (68%, 61%, and 69%, respectively).*GRAPPA* generalized autocalibrating partially parallel acquisitions, *REGAIN* resolution enhancement generative adversarial inline neural network

Three readers, who subjectively assessed each scan, also evaluated 300 short-axis scans for diagnostic quality. In 1.8-fold GRAPPA reconstructed images, 70%, 59%, and 65% of scans were graded as having diagnostic quality (score = 1) (Fig. 8). These percentages remained consistent in 3.3-fold REGAIN-processed images, with 68%, 61%, and 69%, respectively, as evaluated by the three readers. Furthermore, at 5.7-fold acceleration, REGAIN-processed images showed an increased number of diagnostic cases compared with low-resolution counterparts (reader 1: 55% vs 13%, reader 2: 37% vs 24%, and reader 3: 60% vs 54%).

**Fig. 9:**
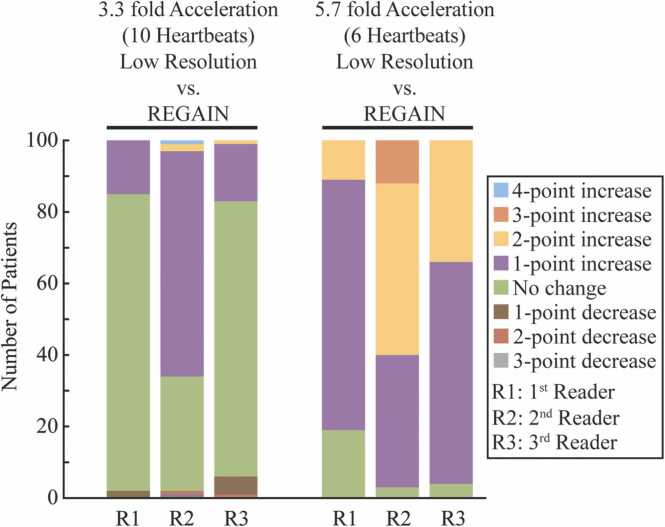
Change in diagnostic scores of blurriness with REGAIN. The stacked bar chart illustrates the number of changes in the reader scores between the low-resolution scan and its REGAIN processes counterpart. Bar colors denote cases with increased (purple: 1-point, yellow: 2-point, orange: 3-point, and blue: 4-point), decreased (brown: 1-point, red: 2-point, and gray: 3-point), or unchanged (green) blurriness. REGAIN reduces blurriness in both 3.3-fold and 5.7-fold accelerations. *REGAIN* resolution enhancement generative adversarial inline neural network

The REGAIN image enhancement resulted in an overall sharpness improvement in reader scores (Fig. 9). Across the 3.3-fold accelerated images and their REGAIN-processed versions, there were 15, 63, and 16 instances of a 1-point improvement, according to the three readers, respectively. Similarly, between the 5.7-fold accelerated images and their REGAIN-processed counterparts, 70, 37, and 62 cases showed a 1-point improvement according to the three readers, respectively. Additionally, there was an overall 18% improvement in image sharpness between 3.3-fold REGAIN-processed images and 1.8-fold GRAPPA reconstructed images (*P* < 0.01, 95% CI: 0.12–0.24) (Table 4). This decreased to 12% (*P* < 0.01, CI: 0.07–0.17) when comparing 5.7-fold accelerated images processed with REGAIN and 1.8-fold accelerated images reconstructed with GRAPPA. Moreover, REGAIN-processed images at 3.3-fold and 5.7-fold acceleration showed an overall 33% (*P* < 0.01, CI: 0.28–0.37) and 91% (*P* < 0.01, CI: 0.88–0.95) improvement in image sharpness compared to their low-resolution counterparts. The percent improvement in image sharpness for each reader is independently reported in Table 4.

**Table 4:** Percent improvement in image sharpness by reader (n = 100).

	3.3-fold acceleration		5.7-fold acceleration	
	Low resolution at 1.5 × 3 mm^2^ vs REGAIN	GRAPPA at 1.8-fold vs REGAIN	Low resolution at 1.5 × 6 mm^2^ vs REGAIN	GRAPPA at 1.8-fold vs REGAIN
Overall				
%	0.33	0.18	0.91	0.12
*P* value	<0.01	<0.01	<0.01	<0.01
95% CI	0.28–0.37	0.12–0.24	0.88–0.95	0.07–0.17
First reader				
%	0.15	0.24	0.81	0.14
*P* value	<0.01	<0.01	<0.01	<0.01
95% CI	0.07–0.23	0.16–0.32	0.76–0.86	0.08–0.20
Second reader				
%	0.66	0.11	0.97	0.09
*P* value	<0.01	<0.01	<0.01	<0.01
95% CI	0.58–0.74	0.03–0.19	0.92–1.02	0.03–0.15
Third reader				
%	0.17	0.19	0.96	0.13
*P* value	<0.01	<0.01	<0.01	<0.01
95% CI	0.09–0.25	0.11–0.27	0.91–1.01	0.07–0.19

*CI* confidence interval, *GRAPPA* generalized autocalibrating partially parallel acquisitions, *REGAIN* resolution enhancement generative adversarial inline neural network

**Fig. 10:**
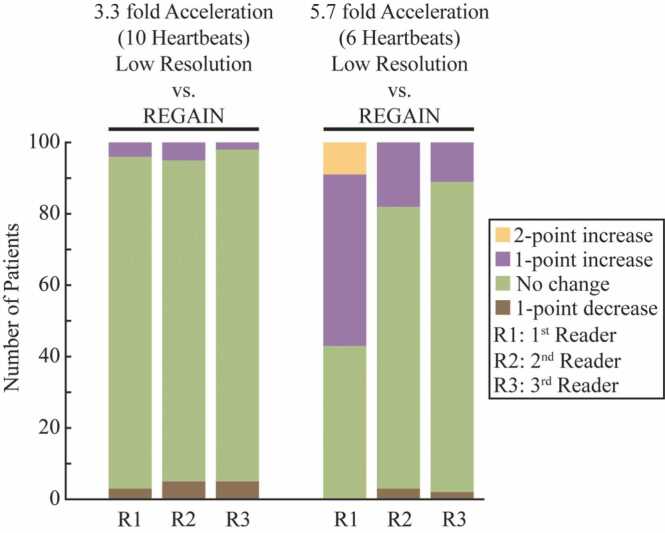
Change in diagnostic quality scores with REGAIN. The stacked bar chart illustrates the number of changes in the reader scores between the low-resolution scan and its REGAIN processes counterpart. Bar colors denote cases with increased (purple: 1-point, yellow: 2-point), decreased (brown: 1-point), or unchanged (green). REGAIN increases diagnostic quality at 5.7-fold acceleration. *REGAIN* resolution enhancement generative adversarial inline neural network

The REGAIN enhancement of LGE images improved image quality at 5.7-fold acceleration, as observed in reader scores (Fig. 10). For the 3.3-fold accelerated images and their REGAIN-processed counterparts, there were no changes in diagnostic quality, with 93%, 90%, and 93% instances of no change (green) scored by the three readers, respectively. In contrast, for the 5.7-fold accelerated images and their REGAIN-processed versions, 48, 18, and 11 cases were demonstrating a 1-point improvement (purple) according to the three readers, respectively. Additionally, there was an overall 11% improvement in diagnostic quality between 3.3-fold REGAIN-processed images and 1.8-fold GRAPPA reconstructed images (*P* < 0.01, confidence interval [CI]: 0.06–0.16) (Table 5). Similarly, there was an 11% improvement when comparing 5.7-fold accelerated images processed with REGAIN and 1.8-fold accelerated images reconstructed with GRAPPA (*P* < 0.01, CI: 0.06–0.17). Moreover, REGAIN-processed images at 3.3-fold only showed a 4% improvement compared with their low-resolution counterparts (*P* = <0.01, CI: 0.02–0.06). This increased to 29% when REGAIN-processed images at 5.7-fold acceleration were compared with their low-resolution counterparts (*P* < 0.01, CI: 0.24–0.33). The percent improvements independently for three readers were shown in Table 5. Additional correlation analysis between perceived improvements in diagnostic utility and image quality is given in Additional file 1: Table S2, where none of the readers or acceleration factors demonstrated a strong correlation.

**Table 5:** Percent improvement in image sharpness by reader (n = 100).

	3.3-fold acceleration		5.7-fold acceleration	
	Low resolution at 1.5 × 3 mm^2^ vs REGAIN	GRAPPA at 1.8-fold vs REGAIN	Low resolution at 1.5 × 6 mm^2^ vs REGAIN	GRAPPA at 1.8-fold vs REGAIN
Overall				
%	0.04	0.11	0.29	0.11
*P* value	<0.01	<0.01	<0.01	<0.01
95% CI	0.02–0.06	0.06–0.16	0.24–0.33	0.06–0.17
First reader				
%	0.04	0.11	0.57	0.1
*P* value	0.04	<0.01	<0.01	<0.01
95% CI	0–0.08	0.05–0.17	0.49–0.65	0.04–0.16
Second reader				
%	0.05	0.13	0.18	0.13
*P* value	<0.01	<0.01	<0.01	<0.01
95% CI	0.01–0.09	0.07–0.19	0.10–0.26	0.07–0.19
Third reader				
%	0.02	0.09	0.11	0.11
*P* value	0.29	<0.01	<0.01	<0.01
95% CI	−0.02–0.06	0.03–0.15	0.03–0.19	0.05–0.17

*CI* confidence interval, *GRAPPA* generalized autocalibrating partially parallel acquisitions, *REGAIN* resolution enhancement generative adversarial inline neural network

Table 6 shows ICC analysis for perceived blurriness and diagnostic quality. For perceived blurriness, the second reader exhibits significant differences compared to the first reader (ICC = 0.50) and moderate agreement with the third reader (ICC = 0.51). Meanwhile, the first and third readers show no significant differences and have good agreement (ICC = 0.72). In terms of diagnostic quality, all three readers show no significant differences and demonstrate excellent agreement (ICC = 0.84, 0.85, and 0.85, respectively).

**Table 6:** Interclass correlation coefficient (ICC) between readers.

	ICC	95% CI
Perceived blurriness		
Readers 1 and 2	0.50	−0.18–0.77
Readers 1 and 3	0.72	0.53–0.83
Readers 2 and 3	0.51	0.06–0.72
Diagnostic quality		
Readers 1 and 2	0.84	0.76–0.89
Readers 1 and 3	0.85	0.76–0.90
Readers 2 and 3	0.85	0.72–0.91

*CI* confidence interval

## 4 Discussion

In this prospective study, we presented a generative AI-based accelerated LGE approach that reduces the scan time without compromising image sharpness. The REGAIN image enhancement, originally trained using cine images, restores image sharpness at both 3.3- and 5.7-fold image acceleration. While there was no significant difference in image quality based on objective image sharpness and subjective evaluation between 3.3- and 1.8-fold accelerated images, we observed lower-quality images acquired with 5.7-fold acceleration.

Image enhancement, image reconstruction, and denoising represent distinct approaches to image acceleration. In image enhancement, AI-based models focus on improving or restoring the spatial resolution of low-resolution images. These models primarily aim to recover missing spatial details in images acquired with low spatial resolution, which are typically free from residual artifacts. Conversely, in image reconstruction-based models, acceleration is achieved through undersampling, violating the Nyquist criteria and resulting in residual aliasing artifacts. AI-based reconstruction models aim to remove these artifacts using k-space-based or image domain-based algorithms. On the other hand, denoising is employed post-acceleration to mitigate noise amplification in residual artifact-free images.

In this study, we used a uniform undersampling, which did not require any sequence modification. CS methods were not compared, as there are currently no 2D LGE sequences available with CS sampling, with most CS trajectories being defined for 3D acquisitions. Additionally, comparisons with other techniques were not included due to the prolonged scan times and the challenges associated with contrast washout.

REGAIN algorithm was developed based on ESRGAIN [56], a super-resolution method for natural image processing. REGAIN incorporates distinct features specifically designed for CMR imaging, such as a single-channel input for grayscale images, the removal of upsampling layers since low-resolution images are generated by zero-padding the acquired low-resolution portion of k-space and a simplified network architecture with fewer parameters. Furthermore, the domain shift between natural images and MRI necessitates training on cine MRI images, preventing a straightforward application. Therefore, we did not compare the performance of REGAIN vs other DL-based super-resolution algorithms.

This study used a REGAIN model pre-trained on cine data rather than a new model trained with accelerated LGE images. Several important issues should be highlighted. One major contributing factor to a robust AI model is the training data. In the original REGAIN study, we implemented a data synthetization pipeline identical to the MRI reconstruction pipeline in the scanner. To enable ease of adoption and facilitate applying the model offline in a vendor-agnostic approach, we chose not to modify the pulse sequence and used a standard GRAPPA acquisition scheme with fewer phase-encoding lines. Synthesizing training data with LGE images is more challenging because the impact of contrast washout in a reduced scan time cannot be synthesized. Furthermore, a model that works across different sequences has more general utility than a sequence-based model. Therefore, we used a trained model using cine for LGE image enhancement. A more dedicated model, trained using LGE, may outperform our approach; however, it is unlikely to be practical to have different models for different sequences. Additional improvements may be achieved by fine-tuning [59] and transfer learning [60], [61] of the original model with additional LGE data, we did not pursue any of these approaches. Further studies of the effect of training datasets in a generative AI model using LGE are warranted. Additionally, REGAIN is implemented inline via FIRE and currently runs on an external NVIDIA DGX-1 server. However, it is not limited to this specific server and can be executed on other graphics processing units (GPUs) or even central processing units (CPUs), although this would result in slower reconstruction times.

Higher image acceleration resulted in shorter breath-hold time. The time saved by REGAIN can be used to acquire multiple slices, improving patient comfort and accommodating patients with difficulty in breath-holding. In this study, we carefully matched the parameters between different scans except for changes in breath-hold time. Additionally, patient motion or respiratory irregularities during prolonged breath holds can induce ghosting artifacts. By reducing acquisition time via REGAIN, motion- or respiratory-induced artifacts can be reduced/eliminated.

In this study, we performed quantitative and subjective image quality assessments to evaluate the performance of REGAIN. Evaluating image sharpness is challenging. Numerous algorithms [62] have been previously used to assess the sharpness of an image, focusing on specific anatomy (e.g., coronary artery). However, there is no robust sharpness measure for evaluating scars. Indeed, scar borders are difficult to delineate, and there are “gray areas” around the scars, reflecting heterogeneous tissue. We used a blur metric for the quantitative analysis of image sharpness [58], while three readers evaluated the degree of blurriness using a 5-level Likert scale. In addition to the subjective assessment of image blurring, we performed an additional assessment to evaluate the diagnostic quality. While there were improvements in diagnostic quality with REGAIN, the resulting scores should be interpreted cautiously. We only acquired a single short-axis slice. Our readers indicated that insufficient coverage and lack of confirmation on scans with different orientations make it challenging to interpret images as diagnostic with confidence; therefore, all three independent readers report difficulties in interpreting LGE images in 1/3 of scans. Further studies using a full LV coverage with companion long-axis images, as performed clinically, should be conducted to evaluate the clinical performance of REGAIN on the confidence of scar interpretation and quantification on LGE.

REGAIN improves upon low-resolution counterparts in terms of blur metric score and perceived blurriness, yet the diagnostic quality remains unchanged. Further rigorous studies in well-defined patient cohorts with scars should be pursued to evaluate if improvements in image sharpness could impact the diagnostic yield or confidence in LGE image interpretation. Furthermore, LGE quantification is highly dependent on image sharpness, and further studies are warranted to study the impact of sharpness improvement on LGE quantification. Furthermore, according to the ICC analyses, the main difference between the readers stems from their varying years of experience, with the second reader having substantially less experience than the other two. Although this difference did not affect the diagnostic quality scores, it had a notable impact on perceived blurriness. Additionally, the first and third readers do not show perfect agreement, highlighting the subjectivity of perceived blurriness.

The data used for the subjective evaluation of perceived image blurriness (Fig. 7) and diagnostic quality (Fig. 8) is the same. However, the number of non-diagnostic cases differs between these assessments. For perceived blurriness, only the impact of image sharpness on diagnosis was considered. In contrast, the diagnostic quality assessment included non-diagnostic cases resulting from various factors, including image artifacts, blurriness, incorrect inversion times, and ghosting artifacts. Therefore, the number of non-diagnostic cases is higher in the diagnostic quality assessment than in the perceived blurriness assessment. Subjective evaluation, particularly in the 5.7-fold accelerated data, suggests an increase in diagnostic quality, as seen with reader 1 (from 13% to 55%). However, the correlation analysis between perceived blurriness and diagnostic quality across all readers and acceleration factors did not reveal a strong relationship. This indicates that perceived blurriness may improve visually, but it does not necessarily correlate with diagnostic accuracy. More comprehensive clinical studies are needed to assess the REGAIN processing on diagnostic outcomes fully.

The utility of REGAIN in enhancing the image quality of cardiac cine images has been rigorously evaluated [50]. This study presented additional data supporting its potential to improve LGE image quality. Despite the initial success, further evaluation in a multi-center study is warranted to evaluate its generalizability across sites carefully. We have tested the feasibility of the REGAIN on other scanners from the same vendor and field strengths for cardiac cine, which showed similar image performance, but further rigorous evaluation is still needed. Additionally, the limited number of subjects with LGE (21 cases) restricts the REGAIN model’s ability to assess performance on scar tissue fully. The pre-trained REGAIN model performs well in sharpening the myocardium and blood pool borders, the main factor contributing to the subjective evaluation in the 79 patients without LGE. However, the results also show that REGAIN enhances scar sharpness in cases with visible scars. A more detailed analysis focusing specifically on LGE cases and structural differences within the scar is warranted.

## 5 Limitations

Our study had several limitations. We only implemented and evaluated the inline model on a Siemens 3T system. However, the model is publicly available and could be tested by other investigators. Second, we did not compare the model's performance vs other AI models. This is a challenging task. There is currently no other model specifically developed for LGE. Different requirements for k-space sampling schemes for various AI models make performing a rigorous comparative study challenging. We only acquired a single mid-left ventricular short-axis slice to make it feasible to perform as an add-on to our clinical patients. We used a >50% threshold for scar transmurality due to the small number of patients with scars in our cohort. Last, scar quantification was not performed due to the limited number of patients with scars and the heterogeneous patient population.

## 6 Conclusion

REGAIN, a generative AI model trained using cine images, improves image sharpness of accelerated 2D LGE images, resulting in reduced scan time. The inline implementation, lack of a need for sequence modification, and publicly available trained model pave the way for independent evaluations for generalizability by other investigators.

## Funding

Reza Nezafat receives grant funding from the 10.13039/100000002National Institutes of Health (NIH) 1R01HL129185, 1R01HL129157, 1R01HL127015, and 1R01HL154744 (Bethesda, Maryland).

## Author contributions

O.B.D. performed data collection, validation, analysis, and manuscript preparation. F.G., C.W.H., and C.W.T. graded image quality. A.C. and L.H.N. were involved in statistical analysis. K.A., J.S., and J.R. were involved with subject recruiting and consenting. T.E.W. and K.C. were involved in the implementation of the inline reconstruction. W.J.M. and R.N. contributed to the study design, validation, data interpretation, and manuscript revision. All authors critically revised the paper and have read and approved the final manuscript.

## Ethics approval and consent

This study was approved by the Beth Israel Deaconess Medical Center Institutional Review Board and was compliant with the Health Insurance Portability and Accountability Act.

## Consent for publication

Written informed consent was obtained from each prospective participant.

## Declaration of competing interest

The authors declare the following financial interests/personal relationships which may be considered as potential competing interests: Reza Nezafat reports financial support was provided by the National Institutes of Health. Tess E. Wallace reports a relationship with Siemens Medical Solutions USA, Inc., Boston, Massachusetts that includes employment. Kelvin Chow reports a relationship with Cardiovascular MR R&D, Siemens Healthcare Ltd., Calgary, Alberta, Canada that includes employment. Reza Nezafat has patent #20240062332 issued to Beth Israel Deaconess Medical Center, Inc. The other authors declare that they have no known competing financial interests or personal relationships that could have appeared to influence the work reported in this paper.

## Data Availability

The proposed reconstruction model is an investigational technique and not available by the vendor as a research tool or product. The model codes are openly available on GitHub: https://github.com/HMS-CardiacMR/REGAIN. Quantitative and qualitative analyses supporting the conclusions of this article are available in the Harvard Dataverse: https://dataverse.harvard.edu/dataverse/cardiacmr, reference number: https://doi.org/10.7910/DVN/VTZVJB.

## Footnotes

Appendix A Supplementary data associated with this article can be found in the online version at doi:10.1016/j.jocmr.2024.101127.
