# Supplementary material for: Late gadolinium enhancement cardiovascular magnetic resonance with generative artificial intelligence
Source: J Cardiovasc Magn Reson. 2024 Nov 28;27(1):101127. doi: 10.1016/j.jocmr.2024.101127 (PMC11761327; doi:10.1016/j.jocmr.2024.101127)
Supplement: Supplementary file 1 — Supplementary material [file mmc1.docx]

**Additional Information**

**REGAIN Generator Architecture**

REGAIN generator network first applies a 3×3 convolution to the input low resolution image, differing from ESRGAN, which employs upsampling layers for image super-resolution. Unlike ESRGAN, where input images are upsampled, in REGAIN, the input images are already zero-padded to the full extent of the desired image resolution. Following this convolution layer, 23 residual dense blocks are used. Each block comprises 3×3 convolutions, followed by a leaky rectified linear unit (LReLU) and concatenation of the previous layer in the channel dimensions, resulting in an increasing channel size up to 320 and a constant output channel size of 64 across 5 inner blocks. All convolutions utilize a 2D kernel size of 3×3, a stride of 1, and 1 padding, while the LReLU activation function has a negative slope of 0.02. After the residual dense blocks, two layers of 3×3 convolutions are utilized, followed by the final layer, which outputs an enhanced image with a single channel. The architecture of the REGAIN generator is illustrated in **Additional file 1: Figure S1A**.

**REGAIN Discriminator Architecture**

The REGAIN discriminator network is comprised of 6 discriminator blocks, each containing two sub-blocks consisting of 3×3 convolutions followed by batch normalization and LReLU activation functions. While the sub-blocks share identical structures, the second sub-block within each discriminator block employs a stride of 2 instead of 1. The number of output channels is doubled at each block, leading to 1024 channels, followed by a final layer of 3×3 convolution. Subsequently, a sigmoid function is employed to compute the relativistic average generative adversarial network loss across the batch of images. All convolutions are 2D with a kernel size of 3×3, a stride of 1, and 1 padding. The LReLU activation function has a negative slope of 0.02. The architecture of the REGAIN discriminator is illustrated in **Additional file 1: Figure S1B**.

**REGAIN Training Details**

REGAIN was trained with Adam optimizer with a learning rate of 0.001, β_1_ = 0.9, and β_2_ = 0.999, using randomly cropped 64×64 image patches and with a batch size of 64 over 75 epochs. The loss function of REGAIN training is given as $\mathcal{L}_{\mathrm{Total}}={10}^{-2}\cdot\mathcal{L}_{\mathrm{pixel}}+ \mathcal{L}_{\mathrm{VGG}}+ {5\cdot10}^{-3}\cdot\mathcal{L}_{\mathrm{GAN}}+ {10}^{-2}\cdot\mathcal{L}_{\mathrm{FTT}}$ where pixel loss is defined as Euclidean distance, visual geometry group (VGG) loss is derived from feature maps of VGG-19 network as perceptual loss, generative adversarial network (GAN) loss is derived from the ESRGAN and lastly fast Fourier transform (FFT) loss is derived from $\mathcal{l}_{1}$ FFT to map images into a spatial frequency domain. The training dataset consists of 1616 participants with short-axis breath-held ECG-gated segmented cine acquisitions covering the whole heart and all cardiac phases. REGAIN was implemented in Python (version 3.7; Python Software Foundation) with the use of the PyTorch library.

**Inline Implementation Details**

A dedicated 32GB memory GPU housed within an NVIDIA DGX-1 server processes the REGAIN reconstruction after receiving the low-resolution images from the FIRE emitter. REGAIN operates with a processing time of ~32 ms. Subsequently, the enhanced images are transmitted to the scanner console through the FIRE injector. The data communication between the scanner and console via FIRE is established using the International Society for Magnetic Resonance in Medicine Raw Data (ISMRMRD) format, with a total transmission time of ~5 milliseconds.

**Blur Metric Details**

We use a no-reference perceptual blur metric [58], which eliminates the need for a reference image and calculates the cost of detail loss due to blurring in the images. This technique is based on our perceptual understanding, where distinguishing between a blurred image and the same re-blurred image is challenging. When a high-resolution image undergoes low-pass filtering, the disparity between the resultant low-resolution image and the original high-resolution image is significant. However, upon re-blurring the low-resolution image, the disparity between the resulting two low-resolution images is minimal. Furthermore, this indicates when a sharp image is blurred, neighboring pixels undergo substantial variation. Conversely, when an already blurred image is further blurred, neighboring pixels experience only minor changes. Therefore, this blur estimation principle [58] entails blurring the initial image and analyzing the variations in neighboring pixels.

First, the input image (**I** $\in\mathbb{R}^{M\times N}$) is blurred with a vertical and horizontal low-pass filter (**H**=$\frac{1}{9}[1 1 1 1 1 1 1 1 1]$, H^T^ = transpose(H)). The resulting images Blurred_Vertrical_ (**B**_Vertical_) and Blurred _Horizontal_ (**B**_Horizontal_) and the input image are then passed to study the variations of neighboring pixels as the absolute differences in images:

|  | $\mathrm{Variation}\mathbf{I}_{\mathrm{Vertical}}(x,y)= \sum_{x=1}^{M-1} \sum_{y=0}^{N-1} \vert\mathbf{I}\left( x,y \right)-\mathbf{I}(x-1,y)\vert,$ | (1) |
| --- | --- | --- |
|  | $\mathrm{Variation}\mathbf{I}_{\mathrm{Horizontal}}\left( x,y \right)= \sum_{x=0}^{M-1} \sum_{y=1}^{N-1} \left\vert\mathbf{I}\left( x,y \right)-\mathbf{I}\left( x,y-1 \right) \right\vert,$ | (2) |
|  | $\mathrm{Variation}\mathbf{B}_{\mathrm{Vertical}}\left( x,y \right)= \sum_{x=1}^{M-1} \sum_{y=0}^{N-1} \left\vert\mathbf{B}_{\mathrm{Vertical}}\left( x,y \right)-\mathbf{B}_{\mathrm{Vertical}}\left( x-1,y \right) \right\vert,$ | (3) |
|  | $\mathrm{Variation}\mathbf{B}_{\mathrm{Horizontal}}\left( x,y \right)= \sum_{x=0}^{M-1} \sum_{y=1}^{N-1} \left\vert\mathbf{B}_{\mathrm{Horizontal}}\left( x,y \right)-\mathbf{B}_{\mathrm{Horizontal}}\left( x,y-1 \right) \right\vert.$ | (4) |

Then, we need to analyze the variations of the neighboring pixels after the blurring compared to the input which is evaluated only on the absolute differences as follows:

|  | $\mathrm{Difference}\mathbf{VB}_{\mathrm{Vertical}}(x,y)= \sum_{x=1}^{M-1} \sum_{y=1}^{N-1} max(0,\mathbf{I}\left( x,y \right)-\mathbf{B}_{\mathrm{Vertical}}(x,y)),$ | (5) |
| --- | --- | --- |
|  | $\mathrm{Difference}\mathbf{VB}_{\mathrm{Horizontal}}(x,y)= \sum_{x=1}^{M-1} \sum_{y=1}^{N-1} max(0,\mathbf{I}\left( x,y \right)-\mathbf{B}_{\mathrm{Horizontal}}(x,y)),$ | (6) |

Here, **DVB_Vertical_** abbreviation is used for the result of Equation 5 showing the difference of the variations of the blurred vertical image and **DVB_Horizontal_** abbreviation is used for the result of Equation 6 showing the difference of the variations of the blurred horizontal image. Then to compare the variations, the sum of coefficients was calculated as follows:

|  | $Sum of Coefficients \mathbf{V}\mathbf{I}_{\mathrm{Vertical}}(x,y)= \sum_{x=1}^{M-1} \sum_{y=1}^{N-1} \mathbf{V}\mathbf{I}_{\mathrm{Vertical}}\left( x,y \right),$ | (7) |
| --- | --- | --- |
|  | $Sum of Coefficients \mathbf{V}\mathbf{I}_{\mathrm{Horizontal}}\left( x,y \right)= \sum_{x=1}^{M-1} \sum_{y=1}^{N-1} \mathbf{V}\mathbf{I}_{\mathrm{Horizontal}}\left( x,y \right),$ | (8) |
|  | $Sum of Coefficients \mathbf{VD}\mathbf{B}_{\mathrm{Vertical}}\left( x,y \right)= \sum_{x=1}^{M-1} \sum_{y=1}^{N-1} \mathbf{DV}\mathbf{B}_{\mathrm{Vertical}}\left( x,y \right),$ | (9) |
|  | $Sum of Coefficients \mathbf{V}\mathbf{DB}_{\mathrm{Horizontal}}\left( x,y \right)= \sum_{x=1}^{M-1} \sum_{y=1}^{N-1} \mathbf{DV}\mathbf{B}_{\mathrm{Horizontal}}\left( x,y \right).$ | (10) |

Subsequently, these results are normalized to range from 0 to 1:

|  | $Blur Vertical=\frac{\mathbf{SV}\mathbf{I}_{\mathrm{Vertical}}\left( x,y \right)-\mathbf{SV}\mathbf{DB}_{\mathrm{Vertical}}(x,y)}{\mathbf{SV}\mathbf{I}_{\mathrm{Vertical}}(x,y)},$ | (11) |
| --- | --- | --- |
|  | $Blur Horizontal=\frac{\mathbf{SV}\mathbf{I}_{\mathrm{Horizontal}}\left( x,y \right)-\mathbf{SV}\mathbf{DB}_{\mathrm{Horizontal}}(x,y)}{\mathbf{SV}\mathbf{I}_{\mathrm{Horizontal}}(x,y)}.$ | (12) |

Finally, the blur metric is defined as the maximum blur annoyance between the vertical and horizontal values as follows:

|  | $Blur=\max\left( Blur Vertical,Blur Horizontal \right),$ | (13) |
| --- | --- | --- |

where no-reference perceptual blur estimation ranges from 0 to 1, where 0 represents the best quality in terms of blur perception, indicating minimal perceived blur, and 1 represents the worst quality, indicating significant perceived blur. All the steps above are depicted in **Additional file 1: Figure S2A and S2B** for a high-resolution LGE input and a low-resolution LGE input, respectively.

If we compare the difference in variations between the blurred images (Difference **VI** - **VB**) and the variations of the input images (**VI**) for both vertical and horizontal images, we observe the differences are less negligible in the high-resolution input (**Figure S2A**) compared to **(Figure S2B)**, where the difference is higher. This higher disparity indicates a greater error in the sum of coefficients, resulting in a higher blur metric, which in turn suggests blurrier images or loss of sharpness.

|  | **Table S1: Transmural Assessment Details** | | | | | | | | | |  |
| --- | --- | --- | --- | --- | --- | --- | --- | --- | --- | --- | --- |
|  |  | 1.8-fold  Acceleration  GRAPPA  1.5 × 1.5 mm^2^ |  | 3.3-fold  Acceleration  Low Resolution  1.5 × 3 mm^2^ |  | 3.3-fold  Acceleration  REGAIN  1.5 × 1.5 mm^2^ |  | 5.7-fold  Acceleration  Low Resolution  1.5 × 6 mm^2^ |  | 5.7-fold  Acceleration  REGAIN  1.5 × 1.5 mm^2^ |  |
|  | # of cases |  |  |  |  |  |  |  |  |  |  |
|  | **Score 1:** ≤25% | 0 |  | 0 |  | 0 |  | 0 |  | 0 |  |
|  | **Score 2:** 26%-50% | 3 |  | 3 |  | 3 |  | 3 |  | 3 |  |
|  | **Score 3:** >25% | 5 |  | 5 |  | 5 |  | 5 |  | 5 |  |

|  | **Table S2: Correlation between perceived improvements in diagnostic utility and image quality** | | | | | |  |
| --- | --- | --- | --- | --- | --- | --- | --- |
|  |  | Reader 1 |  | Reader 2 |  | Reader 3 |  |
|  | **Low-resolution vs. REGAIN at 3.3-fold** |  |  |  |  |  |  |
|  | R/R^2^ | 0.59/0.35 |  | 0.48/0.23 |  | 0.25/0.06 |  |
|  | Regression Equation | **y** = 0.6$*$**x** – 0.89 |  | **y** = 0.54$*$**x** – 1.90 |  | **y** = 0.37$*$**x** – 1.72 |  |
|  | **Low-resolution vs. REGAIN at 5.7-fold** |  |  |  |  |  |  |
|  | R and R^2^ | 0.55/0.31 |  | 0.4/0.16 |  | 0.54/0.29 |  |
|  | Regression Equation | **y** = 0.72$*$**x** – 0.07 |  | **y** = 0.86$*$**x** – 0.66 |  | **y** = 1.09$*$**x** – 0.23 |  |

**
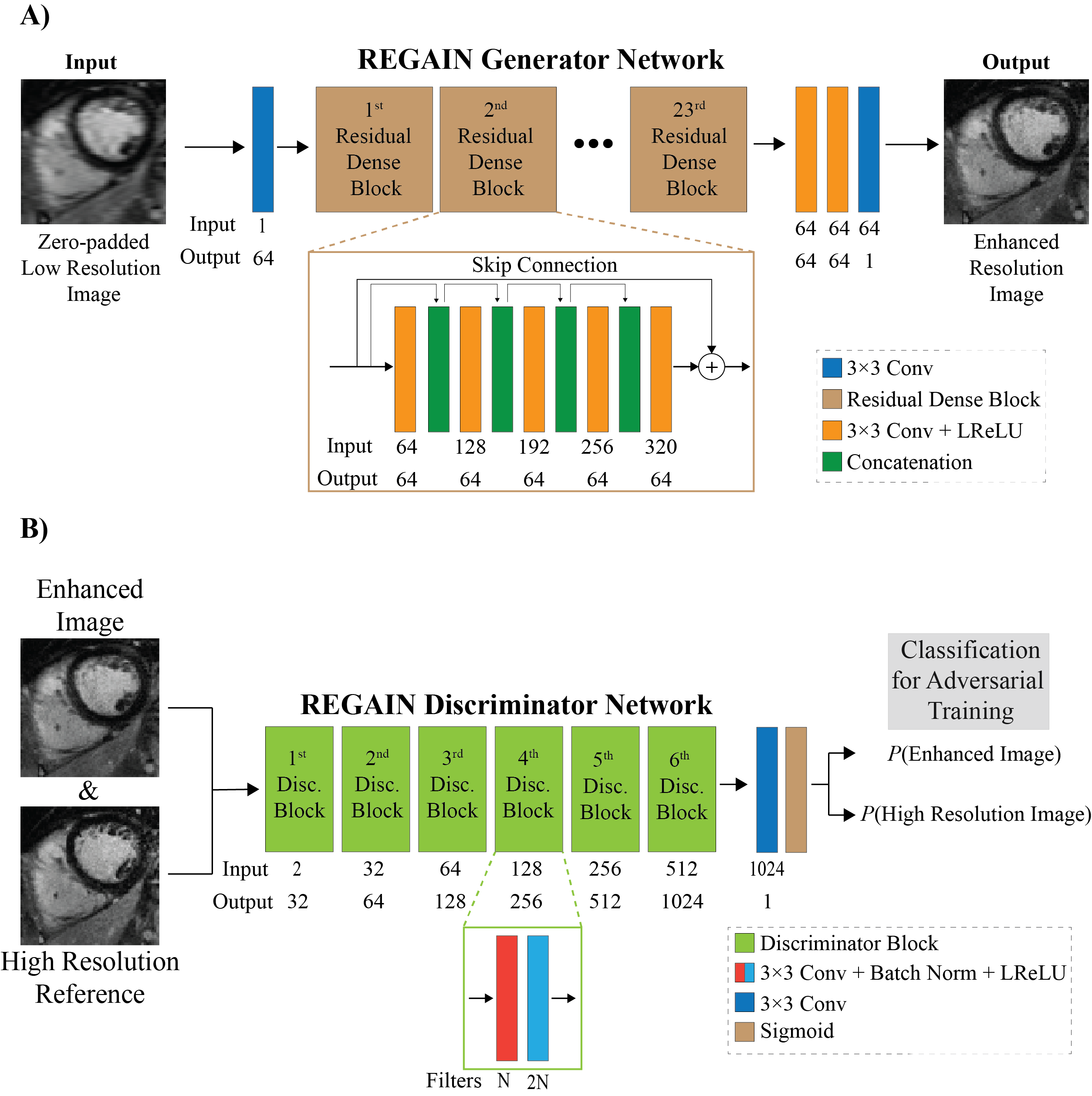
Additional file 1: Figure S1A:** The REGAIN generator and discriminator networks. **(A)** REGAIN generator network consists of 23 residual dense blocks, each having 3×3 convolutions, leaky rectified linear unit (LReLU), and concatenation of the features in channel dimension. **(B)** REGAIN discriminator is used for classification for adversarial training and consists of 6 discriminator blocks with increasing channels using 3×3 convolutions, batch normalization, and LReLU function.

**
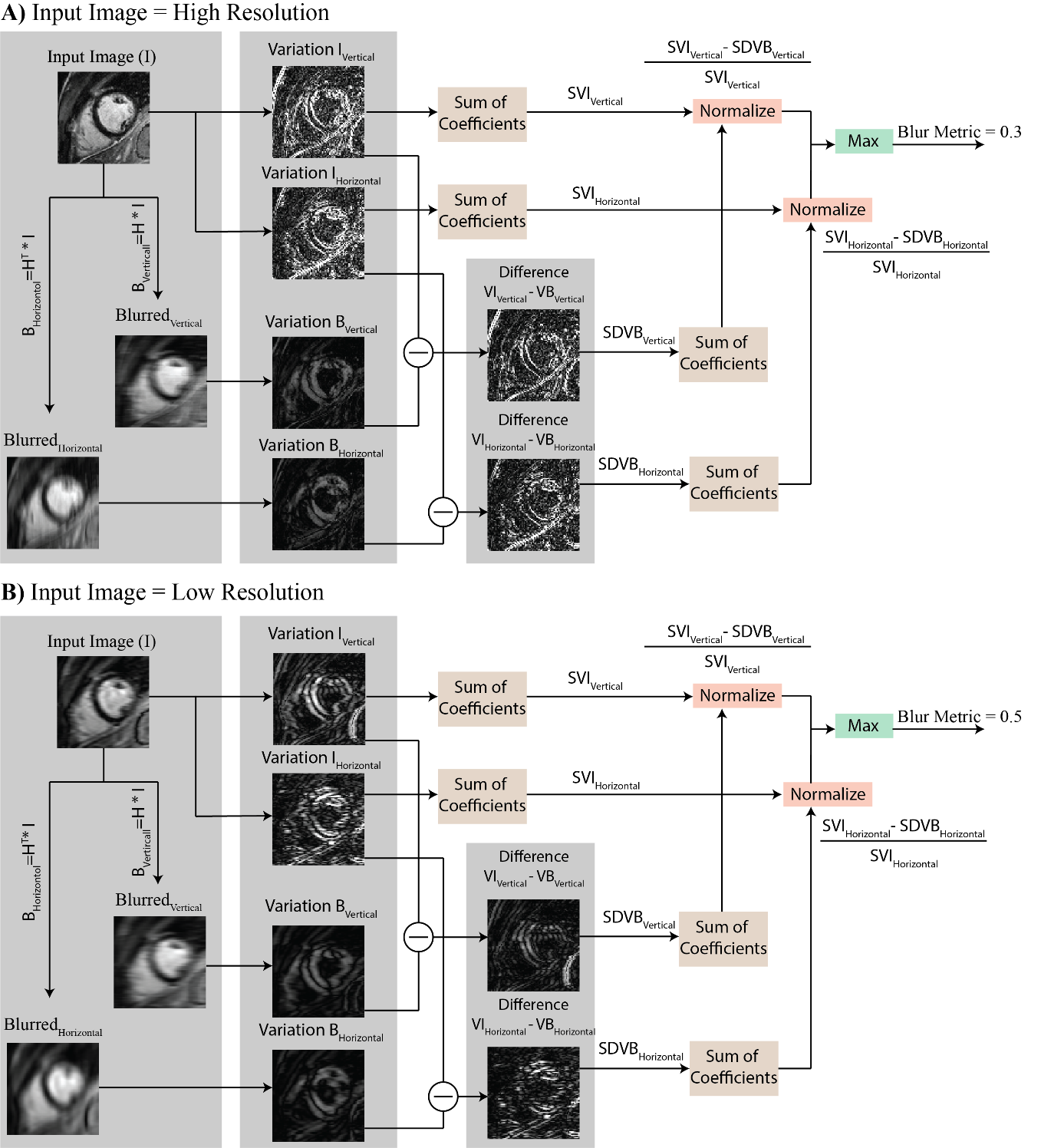
Additional file 1: Figure S2A:** Calculation of the no-reference blur metric. (A) A high-resolution input image leads to a smaller difference between **VI** and a difference between **VI** and **VB** which results in a smaller blur metric of 0.3. (B) Low-resolution input shows a higher amount of difference between VI and the difference between VI and VB, which results in a higher blur metric of 0.5. These indicate the image in (A) is sharper compared to the image in (B).
